# Phase I/II intra-patient dose escalation study of vorinostat in children with relapsed solid tumor, lymphoma, or leukemia

**DOI:** 10.1186/s13148-019-0775-1

**Published:** 2019-12-10

**Authors:** Cornelis M. van Tilburg, Till Milde, Ruth Witt, Jonas Ecker, Thomas Hielscher, Angelika Seitz, Jens-Peter Schenk, Juliane L. Buhl, Dennis Riehl, Michael C. Frühwald, Arnulf Pekrun, Claudia Rossig, Regina Wieland, Christian Flotho, Uwe Kordes, Bernd Gruhn, Thorsten Simon, Christin Linderkamp, Felix Sahm, Lenka Taylor, Angelika Freitag, Jürgen Burhenne, Kathrin I. Foerster, Andreas D. Meid, Stefan M. Pfister, Irini Karapanagiotou-Schenkel, Olaf Witt

**Affiliations:** 1 KiTZ Clinical Trial Unit, Hopp Children’s Cancer Center Heidelberg (KiTZ), German Cancer Research Center (DKFZ) and Heidelberg University Hospital, Im Neuenheimer Feld 430, 69120 Heidelberg, Germany; 2 Department of Pediatric Oncology, Hematology and Immunology, Heidelberg University Hospital, Heidelberg, Germany; 3 Clinical Cooperation Unit Pediatric Oncology, German Cancer Research Center (DKFZ) and German Cancer Consortium (DKTK), Heidelberg, Germany; 4 Division of Biostatistics, German Cancer Research Center (DKFZ) and German Consortium for Translational Cancer Research (DKTK), Heidelberg, Germany; 5 Division of Neuroradiology, Heidelberg University Hospital, Heidelberg, Germany; 6 Division of Pediatric Radiology, Heidelberg University Hospital, Heidelberg, Germany; 7 Faculty of Biosciences, Heidelberg University, Heidelberg, Germany; 8 DKTK Immune Monitoring Unit, German Cancer Research Center (DKFZ) and National Center for Tumor Diseases (NCT), Heidelberg, Germany; 9 Swabian Children’s Cancer Center, University Children’s Hospital Augsburg, Augsburg, Germany; 10 Children’s Hospital, Bremen, Germany; 11 Department of Pediatric Hematology and Oncology, University Children’s Hospital Muenster, Muenster, Germany; 12 Department of Pediatric Oncology and Hematology, Essen University Hospital, Essen, Germany; 13 Division of Pediatric Oncology and Hematology, Freiburg University Hospital, Freiburg, Germany; 14 Department of Pediatric Hematology and Oncology, University Medical Center Eppendorf, Hamburg, Germany; 15 Department of Pediatrics, Jena University Hospital, Jena, Germany; 16 Department of Pediatric Oncology and Hematology, Cologne University Hospital, Cologne, Germany; 17 Department of Pediatric Oncology and Hematology, Hannover University Hospital, Hanover, Germany; 18 Clinical Cooperation Unit Neuropathology, German Cancer Research Center (DKFZ) and German Consortium for Translational Cancer Research (DKTK), Heidelberg, Germany; 19 Department of Neuropathology, Institute of Pathology, Heidelberg University Hospital, Heidelberg, Germany; 20 Pharmacy Department, Heidelberg University Hospital, Heidelberg, Germany; 21 NCT Trial Center, National Center for Tumor Diseases, German Cancer Research Center (DKFZ), Heidelberg, Germany; 22 Department of Clinical Pharmacology and Pharmacoepidemiology, Heidelberg University Hospital, Heidelberg, Germany; 23 Division of Pediatric Neurooncology, German Cancer Research Center (DKFZ) and German Cancer Consortium (DKTK), Heidelberg, Germany

**Keywords:** Vorinostat, Intra-patient dose escalation, Dose-response, Cytokine, Child, HDAC

## Abstract

**Background:**

Until today, adult and pediatric clinical trials investigating single-agent or combinatorial HDAC inhibitors including vorinostat in solid tumors have largely failed to demonstrate efficacy. These results may in part be explained by data from preclinical models showing significant activity only at higher concentrations compared to those achieved with current dosing regimens. In the current pediatric trial, we applied an intra-patient dose escalation design.

The purpose of this trial was to determine a safe dose recommendation (SDR) of single-agent vorinostat for intra-patient dose escalation, pharmacokinetic analyses (PK), and activity evaluation in children (3–18 years) with relapsed or therapy-refractory malignancies.

**Results:**

A phase I intra-patient dose (de)escalation was performed until individual maximum tolerated dose (MTD). The starting dose was 180 mg/m^2^/day with weekly dose escalations of 50 mg/m^2^ until DLT/maximum dose. After MTD determination, patients seamlessly continued in phase II with disease assessments every 3 months. PK and plasma cytokine profiles were determined. Fifty of 52 patients received treatment. *n* = 27/50 (54%) completed the intra-patient (de)escalation and entered phase II. An SDR of 130 mg/m^2^/day was determined (maximum, 580 mg/m^2^/day). *n* = 46/50 (92%) patients experienced treatment-related AEs which were mostly reversible and included thrombocytopenia, fatigue, nausea, diarrhea, anemia, and vomiting. *n* = 6/50 (12%) had treatment-related SAEs. No treatment-related deaths occurred. Higher dose levels resulted in higher C_max_. Five patients achieved prolonged disease control (> 12 months) and showed a higher C_max_ (> 270 ng/mL) and MTDs. Best overall response (combining PR and SD, no CR observed) rate in phase II was 6/27 (22%) with a median PFS and OS of 5.3 and 22.4 months. Low levels of baseline cytokine expression were significantly correlated with favorable outcome.

**Conclusion:**

An SDR of 130 mg/m^2^/day for individual dose escalation was determined. Higher drug exposure was associated with responses and long-term disease stabilization with manageable toxicity. Patients with low expression of plasma cytokine levels at baseline were able to tolerate higher doses of vorinostat and benefited from treatment. Baseline cytokine profile is a promising potential predictive biomarker.

**Trial registration:**

ClinicalTrials.gov, NCT01422499. Registered 24 August 2011,

## Background

Children and adolescents with relapsed or therapy-refractory malignancies have low chances of cure [1–3], implying an unmet clinical need for new therapies [4]. Histone deacetylase inhibitors (HDACi) such as vorinostat, panobinostat, belinostat, romidepsin, and entinostat have shown promising anti-tumor activity in preclinical models and adult clinical trials particularly in leukemia [5]. Vorinostat was the first HDACi to be FDA-approved for the treatment of adult patients with cutaneous T-cell lymphoma with persistent or recurrent disease on or following systemic cytotoxic therapies [6]. Vorinostat is an oral pan-HDACi targeting a broad range of HDACs including HDAC1, HDAC2, and HDAC3 (Class I) and HDAC6 (Class IIb) [7]. Inhibition of HDACs by HDACi induces hyperacetylation of histones and many other cytoplasmic proteins leading to cell cycle arrest, apoptosis, autophagy, and/or cell death.

Several early phase clinical trials using vorinostat as single agent or in combination for various pediatric oncology indications have been performed [8–12]. A pediatric recommended phase 2 dose of 230 mg/m^2^/day was identified for daily and continuous dosing, which approximates the adult dose (adjusted for body surface area) [10]. Substantial variability in pharmacokinetic (PK) parameters was noted, but in general, median AUC and C_max_ appeared to be higher than in adults [13]. In addition, several other combinatorial and non-continuous regimens have been reported (daily dose ranging 180–430 mg/m^2^/day) [8, 9, 11, 12, 14]. Unfortunately, pediatric clinical trials up until now failed to show efficacy [8–12, 14] although individual patients with selected indications clearly benefited from vorinostat treatment [15]. The disappointing clinical results in solid tumors may in part be explained by preclinical data in which vorinostat showed significant anti-tumoral activity only at higher concentrations compared to those achieved with currently applied dosing regimens [16], including neuroblastoma [17] and brain tumor [18] models. At the same time, adult clinical studies suggest that treatment response correlates with dose and a subgroup of patients tolerate doses exceeding the approved dose by up to four-fold [19, 20]. In the current trial, we, therefore, applied an intra-patient dose escalation design in order to maximize the likelihood of response whilst keeping toxicity acceptable for the individual patient.

We report the results of a single-arm phase I/II trial of single-agent vorinostat in children and adolescents with relapsed/refractory solid tumor, lymphoma, or leukemia. The primary objective was to define a safe dose recommendation (SDR) involving a subsequent individual dose escalation regimen. Secondary objectives included PK and to determine tumor response rates, safety, and feasibility. Accompanying exploratory plasma cytokine analyses aimed for predictive biomarker discovery.

## Results

### Patients

**Fig. 1:**
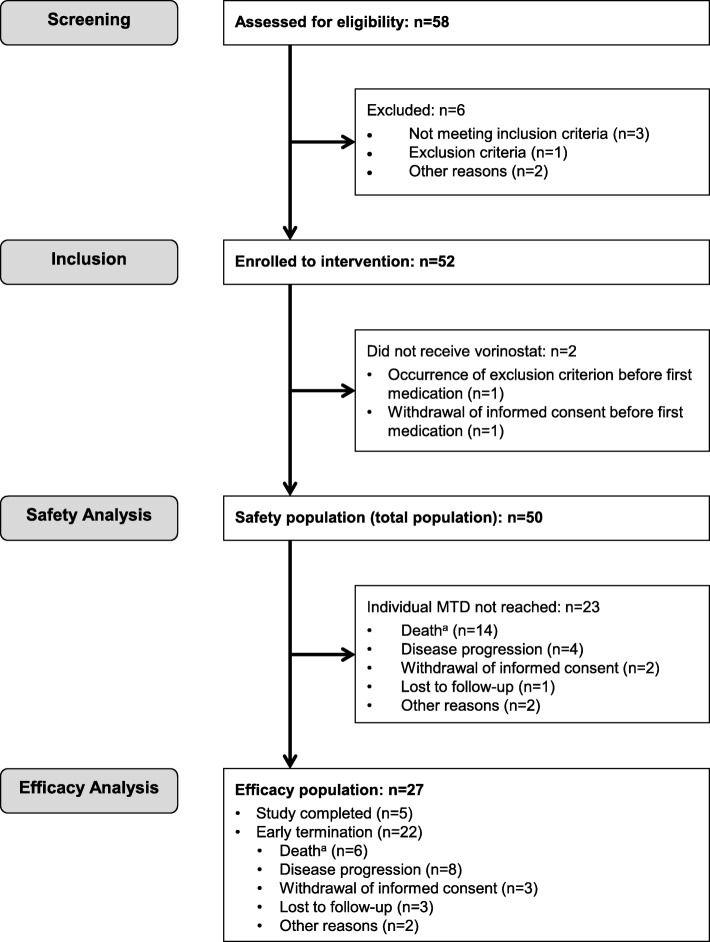
Patient disposition flow diagram. ^a^No treatment related deaths reported

Of the 58 patients screened, 52 were enrolled (three patients did not fulfill inclusion criteria, one had an exclusion criterion, parents refused for one, and one patient died during screening). Two patients were enrolled but did not receive any study medication and were excluded from analysis (in one patient, an exclusion criterion occurred and one had an SAE after which informed consent was withdrawn), resulting in 50 evaluable patients (Fig. 1) (safety population). Patients were enrolled from May 11, 2012, to September 28, 2016. Twenty-eight (56%) patients were male and 22 (44%) female. The age for the total population was 10.9 ± 4.1 years (mean ± SD). The most common diagnoses were brain tumors, followed by sarcomas and several other entities (Table 1).

**Table 1:** Baseline characteristics for eligible patients

Characteristic	Safety population (*n* = 50)	Efficacy population (*n* = 27)
	No (%)	No (%)
Sex		
Female	28 (56)	12 (44)
Male	22 (44)	15 (56)
Age, years, median (range)	11 (3 - 18)	11 (5-17)
Diagnosis		
Brain tumors		
High-grade glioma	19 (38)	11 (41)
Medulloblastoma	8 (16)	4 (15)
Ependymoma	3 (6)	2 (7)
Low grade glioma	2 (4)	0
PNET	1 (2)	1 (4)
Other^a^	1 (2)	1 (4)
Extracranial solid tumors		
Ewing sarcoma	4 (8)	3 (11)
Osteosarcoma	4 (8)	2 (7)
Neuroblastoma	2 (4)	0
DSRCT	2 (4)	1 (4)
Soft tissue sarcoma	1 (2)	1 (4)
Wilms tumor	1 (2)	0
SETTLE tumor	1 (2)	1 (4)
Leukemia		
AML	1 (2)	0
Lansky score		
100	9 (18)	5 (19)
90	13 (27)	8 (30)
80	14 (29)	11 (41)
70	6 (12)	2 (7)
60	7 (14)	1 (4)
Missing	1 (2)	0

Baseline characteristics for eligible patients for both the total population (safety population) at baseline (*n* = 50) and the efficacy population (population which reached their individual MTD: (*n* = 27)), separately

*DSRCT*, desmoplastic small round cell tumor; *SETTLE*, spindle epithelial tumor with thymus like differentiation

^a^Malignant pleomorphic neuroepithelial tumor

Of the 50 patients, 27 (54%) completed the intra-patient (de)escalation period and had at least one visit in the phase II part of the trial receiving vorinostat at their individual MTD (efficacy population). There were no significant demographic and baseline characteristic differences between the safety and efficacy population at baseline (Table 1). There were no neuroblastoma or leukemia patients present in the efficacy population: hence, all tumors were evaluated using RECIST version 1.1.

### Safety

The safety population comprised all 50 patients. The median duration of drug exposure was 102 days (range, 1–485 days). Ten different dose levels were administered ranging from 130 mg/m^2^/day to 580 mg/m^2^/day, most of the patients received three (*n* = 14/50, 28%) or four (*n* = 9/50, 18%) different dose levels. Out of 50, 48 (96%) of patients had a starting dose of 180 mg/m^2^/day and 4/50 (8%) of patients reached the maximum individual MTD of 580 mg/m^2^/day (median individual MTD, 280 mg/m^2^/day) (Additional file 4). An SDR (defined as the highest dose with a DLT in no more than 1/50 patients) as starting dose for subsequent individual dose escalation was 130 mg/m^2^/day. During the study, four patients needed de-escalation to 130 mg/m^2^/day and one of them had DLTs at this dose. There were up to 9 DLTs per patient, but most patients had one (*n* = 12/50, 24%) or two (*n* = 10/50, 20%) DLTs. Most DLTs were related to the blood and lymphatic system, most commonly decreased platelet counts (52 DLTs) followed by decreased white blood cell count (11 DLTs). Most frequent non-hematological DLTs were fatigue (7 DLTs), hyponatremia, and nausea (both four DLTs). Other DLTs mostly concerned gastrointestinal disorders and metabolism and nutrition disorders (e.g., electrolyte disturbances) (Table 2).

**Table 2:** Patients with DLT per dose level (safety population, *n* = 50).

DLT	Patients (%) with DLT per dose level (mg/m^2^/day)										Total sum of DLTs
	130	180	230	280	330	380	430	480	530	580	
Hematological DLTs											
Platelet count decreased		6 (12%)	8 (16%)	13 (26%)	6 (12%)	4 (8%)	4 (8%)	3 (6%)	4 (8%)	3 (6%)	52^a^
White blood cell decreased		1 (2%)	3 (6%)	2 (4%)	2 (4%)	1 (2%)	1 (2%)			1 (2%)	11
Anemia		1 (2%)								1 (2%)	2
Non-hematological DLTs											
Fatigue	1 (2%)	1 (2%)	1 (2%)	1 (2%)			1 (2%)	1 (2%)		1 (2%)	7
Hyponatremia		2 (4%)			1 (2%)		1 (2%)				4
Nausea	1 (2%)	1 (2%)	1 (2%)	1 (2%)							4
Alanine aminotransferase increased		1 (2%)	2 (4%)								3
Decreased appetite	1 (2%)	1 (2%)	1 (2%)								3
Hypermagnesemia				1 (2%)	1 (2%)		1 (2%)				3
Abdominal pain upper	1 (2%)	1 (2%)									2
Febrile infection				1 (2%)						1 (2%)	2
Vomiting			1 (2%)	1 (2%)							2
Weight decreased			1 (2%)							1 (2%)	2
Abdominal pain				1 (2%)							1
Aggression		1 (2%)									1
Apathy		1 (2%)									1
Hypokalemia			1 (2%)								1
Loss of personal independence in daily activities							1 (2%)				1

Patients can have several DLTs at the same time at the same dose level. Furthermore, due to the intra-patient dose (de-)escalation design, patients could be treated in several dose levels, resulting in more DLTs at respective dose levels

^a^Including 1 accidental overdose (714 mg/m^2^/day), data not depicted

AEs were considered as treatment related if the relationship was reported as “related,” “probable,” “possible,” or missing. The majority of the patients (*n* = 46/50, 92%) experienced treatment-related AEs, *n* = 6/50 (12%) of them had treatment-related SAEs. A total of *n* = 42/50 (84%) patients experienced severe treatment-related AEs (CTCAE grade 3 or 4) of which most were reversible. *n* = 6/50 (12%) patients discontinued treatment and *n* = 35/50 (70%) patients had dose reductions or temporary discontinuations due to treatment-related AEs (Additional file 5). No treatment-related deaths were reported. The most common treatment-related AE was decreased platelet count (*n* = 37/50 patients, 74%) of which *n* = 15 were grade 3 and 16 grade 4. Other frequent treatment-related AEs (mostly grade CTCAE grades 1–2) were fatigue in *n* = 16/50 (32%), nausea in *n* = 15/50 (30%), diarrhea in *n* = 12/50 (24%), anemia in *n* = 10/50 (20%), and vomiting in *n* = 10/50 (20%) patients (Table 3).

**Table 3:** Treatment related AEs

	Patients (%, 95% CIs) with AE (maximum Grade CTCAE v4.0)			
Adverse event	Grade 1 - 2	Grade 3	Grade 4	Total of patients with AE
Hematological AEs				
Platelet count decreased	6 (12.0)	15 (30.0)	16 (32.0)	37 (74.0, 59.7 - 85.4 )
Anemia	8 (16.0)	2 (4.0)		10 (20.0, 10.0 - 33.7)
White blood cell decreased	1 (2.0)	8 (16.0)		9 (18.0, 8.6 - 31.4 )
Non-hematological AEs				
Fatigue	9 (18.0)	7 (14.0)		16 (32.0, 19.5 - 46.7)
Nausea	11 (22.0)	4 (8.0)		15 (30.0, 17.9 - 44.6)
Diarrhea	11 (22.0)	1 (2.0)		12 (24.0, 13.1 - 38.2)
Vomiting	8 (16.0)	2 (4.0)		10 (20.0, 10.0 - 33.7)
Alopecia	9 (18.0)			9 (18.0, 8.6 - 31.4)
Weight decreased	5 (10.0)	3 (6.0)		8 (16.0, 7.2 - 29.1)
Decreased appetite	5 (10.0)	2 (4.0)		7 (14.0, 5.8 - 26.7)
Headache	5 (10.0)	1 (2.0)		6 (12.0, 4.5 - 24.3)
Blood creatinine increased	5 (10.0)			5 (10.0, 3.3 - 21.8)
Alanine aminotransferase increased	2 (4.0)	2 (4.0)		4 (8.0, 2.2 - 19.2)
Blood lactate dehydrogenase increased	4 (8.0)			4 (8.0, 2.2 - 19.2)
Constipation	4 (8.0)			4 (8.0, 2.2 - 19.2)
Dry skin	4 (8.0)			4 (8.0, 1.3 - 16.5)
Cough	3 (6.0)			3 (6.0, 1.3 - 16.5)
Hypermagnesemia		3 (6.0)		3 (6.0, 1.3 - 16.5)
Pyrexia	3 (6.0)			3 (6.0, 1.3 - 16.5)

Incidence and maximum severity (the maximum grade for every AE per patient is depicted for the duration of the study) of treatment related AEs occurring in at least 5% of patients according to CTCAE v4.0 (safety population, *n* = 50). “Treatment related” was defined as a relationship reported as “related”, “probable” or missing. No treatment related deaths were reported

### Efficacy

The efficacy population consisted of *n* = 27/50 (54%) patients who completed the intra-patient (de)escalation period and had at least one visit in the phase II part of the trial at their individual MTD. *n* = 5/27 (18.5%) patients reached the end of the maximum treatment period of 12 months. The other *n* = 22/27 patients (81.5%) terminated the study prematurely. Reasons for premature termination were death in *n* = 6 (27%), disease progression in *n* = 8 (41%), withdrawal of informed consent in *n* = 3 (14%), lost to follow-up in *n* = 3 (9%), and other reasons in *n* = 2 (9%) cases. The median duration of drug exposure in the efficacy population was 164 days (range, 29–485 days).

**Fig. 2:**
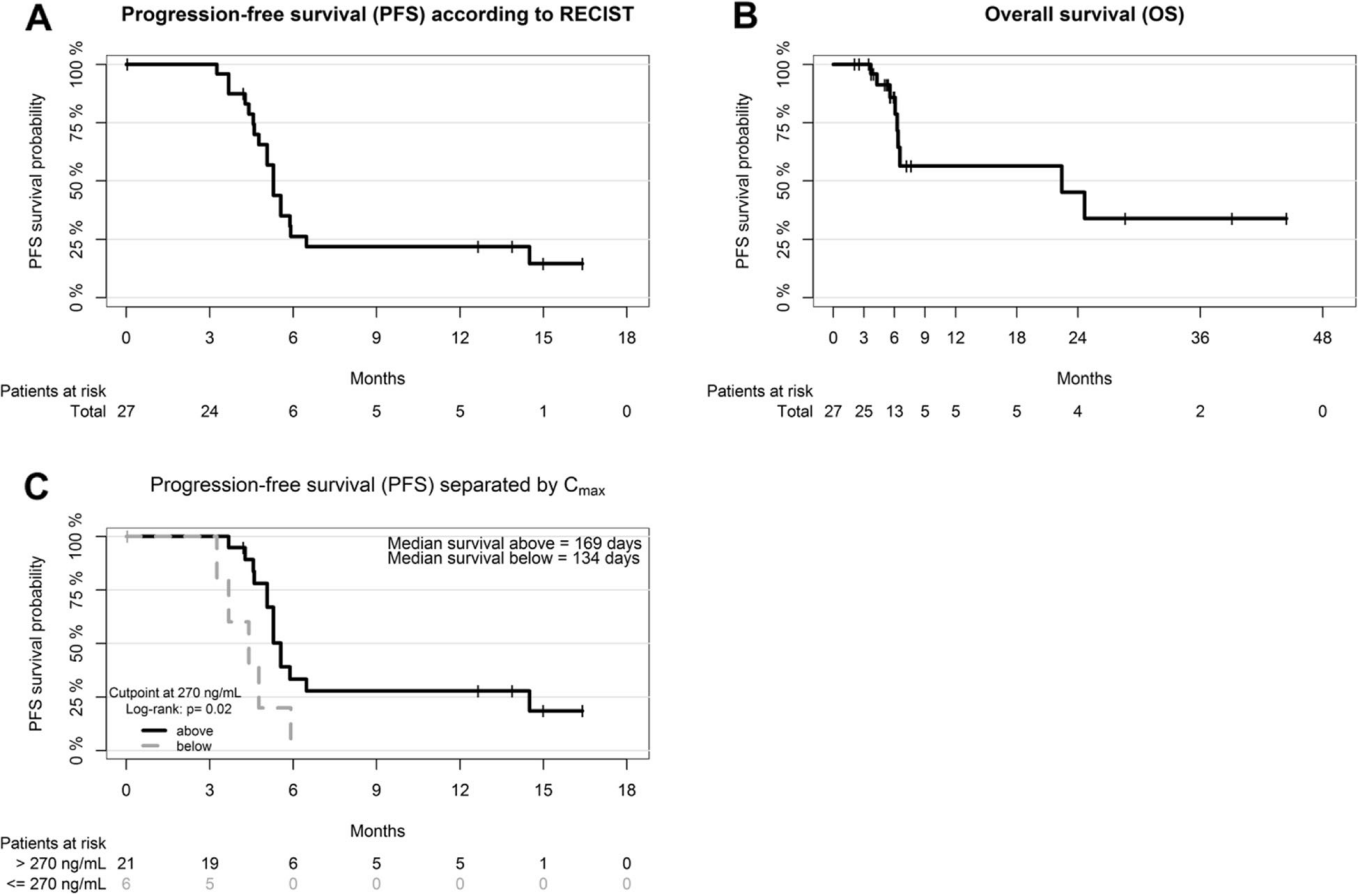
Survival curves**. a** PFS, efficacy population (*n* = 27). b OS, efficacy population (*n* = 27). Including data after end of study. c Kaplan-Meier plot with separation for C_max_ (ng/mL). PFS according to the optimal cutpoint at 270 ng/mL. Log-rank, *p* = 0.02 (exploratory analysis)

At first response evaluation (after 3-month treatment at individual MTD), *n* = 3/27 patients showed partial response (PR, 11%) and *n* = 2/27 showed stable disease (SD, 7%). After 6 months of treatment at individual MTD, an additional PR occurred, resulting in a best overall response rate (combining PR and SD, no CR observed) of 22% (Table 4). The median duration of response was 19.1 months. Median progression-free survival (PFS) and overall survival (OS) were 5.3 and 22.4 months, respectively, for the efficacy population (Table 4 and Fig. 2a, b)

**Table 4:** Efficacy end points

End point	Safety population (*n* = 50)	Efficacy population (*n* = 27)
Best RR (CR + PR), No. (%, 95% CI)	4 (8.0, 2.2–19.2)	4 (14.8, 4.2–33.7)
Best ORR (CR + PR + SD), No. (%, 95% CI)	6 (12.0, 4.5–24.3)	6 (22.2, 8.6–42.3)
Median response duration, months	19.1	19.1
Median PFS, months (95% CI)	4.6 (3.7–5.3)	5.3 (4.6–5.9)
Median OS, months (95% CI)	6.1 (4.0–10.4)	22.4 (6.3 - --)

*RR*, response rate; *CR*, complete response (not observed); *PR*, partial response; *SD*, stable disease; *ORR*, overall response rate; *PFS*, progression free survival; *OS*, overall survival

Of the five patients who reached the end of study (stayed on treatment > 12 months), all had an individual MTD in the highest dose levels and four patients had PR as best response (Table 5). Some of these patients continued treatment for several years beyond the study observation period (not in included in data analysis). Among these were two patients with continued partial response and clinical benefit who received prolonged treatments. One patient with a centrally confirmed histological diagnosis of glioblastoma WHO IV (DNA methylation profile: pilocytic astrocytoma with BRAFV600E mutation) received vorinostat for a total duration of 3.5 years and continued to be in partial remission 1 year after treatment cessation. One patient with a pulmonary metastasized spindle epithelial tumor with thymus-like differentiation (SETTLE) received vorinostat for a total duration of 5 years with continued partial response. Progression occurred after discontinuation of vorinostat.

**Table 5:** Diagnosis, response, survival, dosing and molecular data of long term survivors

Histological diagnosis	Best response	PFS	OS	Last known status	MTD	Last dose level	Methylation profile	Copy number variations	Mutations and variants
		Days	Days		mg/m^2^/d	mg/m^2^/d			
High grade glioma	PR	385 (C)	1352 (C)	Alive	280	280	Pilocytic astrocytoma	-	BRAF (p.V600E)
Medulloblastoma	SD	422 (C)	871 (C)	Alive	580	430	Not attributable; highest score for medulloblastoma group 4	-	-
High grade glioma	PR	441 (NC)	750 (NC)	Deceased	530	530	Not attributable; highest score for glioblastoma IDH wt	Homozygous loss of CDKN2A/B; several gains, e.g. 7p (EGFR), 9q (PTCH), 11q (CCND1); several losses, e.g.: 10q (MGMT)	Mutations: TP53 (p.R174X), ATRX (p.R907X), NF1 (p.R1968X); variants (no germline data available): RET (p.R820H), BCRA2 (p.E2856A), ATR (p.Y1462C), PIK3C2G (p.N1211S), TP53 (p.G113S)
SETTLE tumor	PR	456 (C)	1190 (C)	Alive	580	580	-	No aberrations	Mutations: none; variants: none
High grade glioma	PR	499 (C)	682 (NC)	Deceased	530	280	-	-	-

Overview of patients with partial reponse and/or long term stable disease (reaching > 12 months treatment). *Abbreviations*: *PFS* progression free survival, *OS* overall survival, *MTD* maximal tolerable dose, *(C)* censored, *(NC)* not censored, *SETTLE* spindle epithelial tumor with thymus like differentiation

### Pharmacokinetic studies

PK evaluation was performed on day 8 after start treatment, at the time of reaching the individual MTD and 3 months thereafter (at the time of the first response evaluation). A C_max_ for all ages and dose levels normalized to 1 mg of vorinostat per day (C_max_/D) of 1.70 ± 1.18 ((ng/mL)/(mg/d)), a T_max_ of 2.07 ± 1.37 h and T_1/2_ of 1.98 ± 0.96 h were detected. Table 6 summarizes further PK results for all ages and dose levels. PK data according to dose level is provided in the Additional files 1 and 6. Although there was substantial interpatient variability, C_max_ was higher in the higher dose levels (Additional file 1), whereas for area under the curve (AUC), this was not the case (data not shown). An explorative analysis showed that patients who achieved a higher C_max_ (and thus received higher doses) had longer PFS (Fig. 2c). The five patients who achieved prolonged disease control (> 12 months) all had a C_max_ of > 270 ng/mL with high-range individual MTDs from 280–580 mg/m^2^/day (response, survival, and dosing can be found in Table 5). The tumors of the five patients who achieved prolonged disease control all had different histology (Table 5). Of note, brain tumors were enriched in this group. No relevant influence of age on PK parameters was detected. Explorative analyses did not reveal correlation between most frequently occurring toxicity, i.e., thrombocytopenia, and dose/PK parameters like T_max_ or AUC (data not shown).

**Table 6:** Pharmacokinetic parameters

	C_max_/D	T_max_	AUC0_0-inf_/D	t_½_	Clearance	V_z_
	((ng/mL)/(mg/d))	(h)	((ng/mL*h)/(mg/d))	(h)	(L/h)	(L)
No. of Samples	86	86	66	79	79	79
Mean	1.70 ± 1.18	2.07 ± 1.37	5.49 ± 2.75	1.98 ± 0.96	235 ± 149	644 ± 460

PK parameters (± SD). Means of all ages and dose levels

C_max_/D, maximum concentration normalized to 1 mg vorinostat per day; T_max_, time at maximum concentration; AUC0_inf_/D, area under the curve from 0 to infinitive normalized to 1 mg vorinostat per day; t_½_, half-life time of vorinostat; V_z_, distribution volume

### Biomarker analyses

HDAC inhibitors have been shown to modulate expression of immune-related genes and regulate cytokine production in several preclinical models [21]. Therefore, cytokine profiles in plasma samples of study subjects from which at least two blood samples including the baseline sample were available were determined. A total number of *n* = 119 plasma samples from *n* = 43 patients from a maximum of *n* = 4 time points (baseline, *n* = 43 samples; day 8, *n* = 40 samples; MTD day 1, *n* = 21 samples; and at 3 months after reaching MTD; *n* = 15 samples) were measured by multiplex assay covering 27 cytokines.

**Fig. 3:**
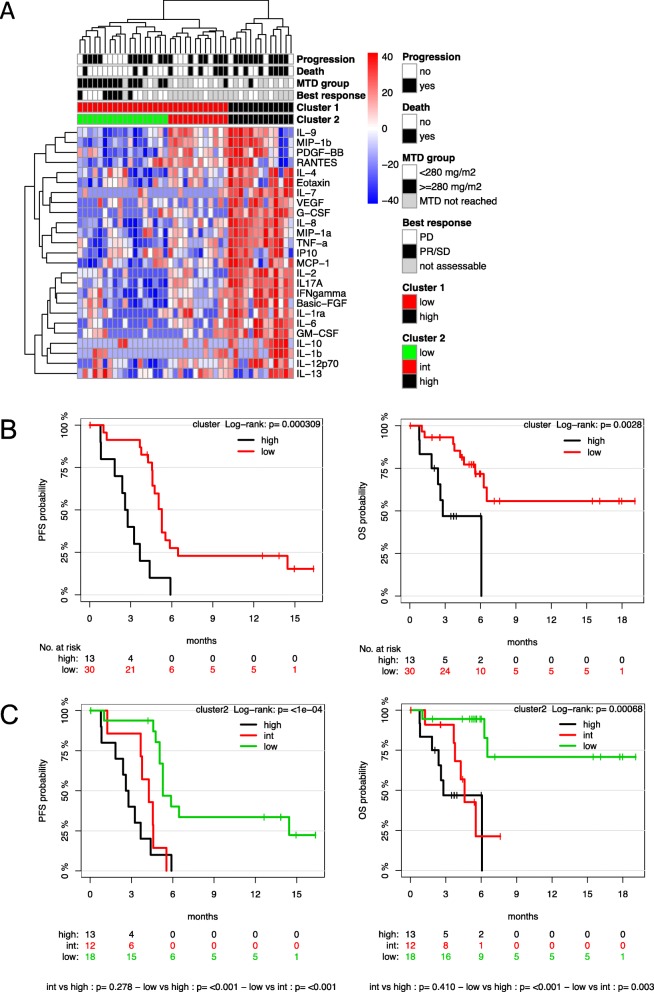
Low levels of cytokine expression at baseline define a cohort with favorable outcome. **a** Heat map of baseline concentrations (Gehan u-score). MTD group, < 280 = MTD reached was below 280 mg/m^**2**^, > = 280 = MTD reached was equal to or above 280 mg/m^**2**^, not reached = MTD was not reached; Best Response: PD: best response progressive disease; PR/SD: best response partial response or stable disease; not assessable: best response not assessable. **b** PFS and OS Kaplan-Meier plot according to cytokine cluster high and low. c PFS and OS Kaplan-Meier plot according to cytokine cluster high, intermediate (int), and low in cluster 2

A high correlation between individual cytokines at baseline was detected (Additional file 2), suggesting a high intra-patient correlation of the measured cytokines. Unsupervised clustering of the cytokine profiles at baseline revealed two separate clusters (1 and 2), with cluster “high” characterized by high and cluster “low” by low cytokine levels (Fig. 3a). Both clusters differed statistically significantly for PFS and OS (*p* < 0.0005 and *p* < 0.005 respectively) (Fig. 3b), with “low” cytokine levels associated with a favorable PFS and OS. Within cluster “low,” two sub-clusters (“low” and “intermediate”) were distinguishable, with statistically different PFS and OS overall (Fig. 3c), again with “low” cytokine levels associated with a favorable PFS and OS. The measurement of 9 cytokines was sufficient to distinguish the three clusters “high,” “intermediate,” and “low” (Additional file 2). On a single factor level, measurement of, e.g., IL8 alone was able to discriminate between the groups “high” and “low” (cluster 1) (Additional file 3), and IL9 and MIP1b in addition discriminated between the groups “high,” “intermediate,” and “low” (cluster 2) (Additional file 3).

Longitudinal analysis of samples revealed no significant differences in mean concentration over time, as detected at the four time points analyzed, separated either by MTD groups (≥ 280 mg/m^2^, < 280 mg/m^2^, or MTD not reached; Additional file 2) or by best response groups (PR/SD, PD, not assessable; Additional file 2) indicating stable cytokine profiles in individual patients. In summary, exploratory biomarker analysis revealed low baseline plasma cytokine levels as indicator of favorable clinical outcome.

## Discussion

Our pediatric phase I/II study of vorinostat in children and adolescents with relapsed/refractory malignancies identified a safe starting dose of 130 mg/m^2^/day for individual dose escalation with weekly increments of 50 mg/m^2^. Although the median individual MTD reached after individual dose escalation was 280 mg/m^2^/day, 4/50 of patients reached the maximum individual MTD of 580 mg/m^2^/day, confirming that higher doses can be tolerated by individual patients, as has been published in earlier adult trials and also observed in a recent study in neuroblastoma [14]. Most common treatment-related AE was decreased platelet count followed by fatigue, nausea, diarrhea, anemia, and vomiting. This AE profile is similar to what has been reported for vorinostat in other pediatric and also adult studies. No treatment-related deaths were reported.

An important limitation of the study design is that the definition of SDR was potentially too rigid. The definition did not account for whether DLTs resolved without severe sequelae after discontinuation (in other words, were manageable), as was the case for the most frequent DLTs, i.e., blood and lymphatic system DLTs. This resulted in a SDR of 130 mg/m^2^/day for individual dose escalation (dose level 1), which may seem contradictory to the median individual MTD of 280 mg/m^2^/day. It seems feasible to start vorinostat treatment at one or two dose levels higher (180 or 230 mg/m^2^/day) without significantly compromising safety as long as patients are closely monitored (blood counts, electrolytes, GI disturbances) and weekly increments of 50 mg/m^2^ are not exceeded.

Exploratory analysis demonstrated that dose escalation to doses higher than the currently recommended pediatric dose [10] of 230 mg/m^2^/day, corresponding to the approved adult dose of 400 mg/day, resulted in higher C_max_ and was associated with tumor response and longer progression free survival. The five patients who achieved prolonged disease control all had a C_max_ of > 270 ng/mL which corresponds to preclinical testing data from pediatric cancer models which showed significant activity only at median IC50 of 1.44 μM (381 ng/mL) [16]. Furthermore, since the HDAC inhibitory activity of vorinostat closely follows the drug concentration due to a short lifetime of the drug-target complex as has been shown in preclinical models [18], it is well conceivable that a higher C_max_ leads to longer inhibition of HDAC. This observation underlines the importance of intra-patient dose escalation of vorinostat as single agent in order to maximize the likelihood of benefit for the patient.

The vorinostat PK data are in the range of previously published pediatric vorinostat PK [10–12]. Investigators in these studies did, however, not increase to doses above 300 mg/m^2^, but also used the doses of 180, 230, and 300 mg/m^2^ and published steady-state AUCs between 817 and 2963 ng/mL*h, which is in line with the range of 752 to 2574 ng/mL*h determined in this study (130–330 mg/m^2^ dosing). With higher dosing (up to 580 mg/m^2^) AUCs up to 3840 ng/mL*h were reached. Further PK data (T_1/2_, T_max_, Cl/F, V_D_) was also comparable [11]. Inter-individual variability of vorinostat in patients is high, which is well known for vorinostat [22–24] due to absorbance variability with different meals or fasted. In comparison to adult data, children exposures calculated as AUC were reported to be substantial higher [19, 23, 25] and could be confirmed by the data in this study. The adults show longer half-lives and the clearance is higher than in children suggesting that tissue distribution in adults is more pronounced [19].

The overall response rate of 22% (combining PR and SD, no CR observed) was lower as reported by trials in comparable populations (46%), whereas the observed OS (median 22.4 months) was longer as reported in comparable populations (9.0 months) [1]. Among the four patients experiencing a partial response, three had a national reference neuropathology diagnosis of high-grade glioma (HGG) suggesting that this may be a target population for future studies as suggested by others [26] and suggesting blood-brain barrier penetrance in agreement with preclinical observations by others [27–29]. Molecular analyses of archived tumor material from primary diagnosis revealed that one of those patients with HGG displayed the molecular features of BRAFV600E-mutated pilocytic astrocytoma. The observation of a responding molecularly defined BRAF-mutated pilocytic astrocytoma is intriguing in light of the recently discovered ability of vorinostat to induce apoptosis by increasing reactive oxygen species (ROS) levels in BRAFV600E-mutated melanomas [30]. A total of two patients with continued PR and clinical benefit tolerated treatment with vorinostat for up to 5 years, indicating long-term tolerability.

Predictive biomarkers for the treatment with HDACis are scarce. Immunohistochemistry for HR23B has been shown to be a positive predictive marker for response to vorinostat [31] and for belinostat [32] in clinical studies. Preclinical studies indicate *MYC* amplification as a potential predictive marker for HDACi treatment [18, 33]. Since several HDAC inhibitors have shown immune-modulatory effects in preclinical models [21, 34] and more recently, to act synergistically with immune checkpoint inhibitors [35–38] we sought to correlate plasma cytokine profiles with clinical outcome in our study. Surprisingly, unsupervised clustering revealed a cohort of patients with favorable outcome defined by low levels of cytokine expression at baseline. Furthermore, all five patients exhibiting partial responses/prolonged stable disease with a favorable clinical outcome showed a low cytokine expression profile at baseline. In contrast, adult phase I/II trials of vorinostat in clear-cell renal cell carcinoma [39] and panobinostat in lymphoma [40, 41] did not detect a difference of baseline cytokine expression profiles between responders and non-responders. Of note, our trial did not enroll patients with any of these tumors and the determined cytokine profiles differed compared to these adult studies. Thus, our data suggests that baseline plasma cytokine levels can potentially serve as predictive biomarker for treatment response and/or improved tolerability to HDACi such as vorinostat in pediatric cancers requiring further prospective investigation. It remains poorly understood by which biological mechanism each single cytokine interacts with vorinostat and the immune system, and how this relates to anti-tumoral activity of vorinostat, which should be addressed in future studies.

### Conclusion

Intra-patient dose escalation of vorinostat in children and adolescents with relapsed/refractory malignancies seems feasible, results in manageable toxicity, and can induce partial responses or disease stabilization in a fraction of patients. Higher dose levels of vorinostat correlate with plasma peak levels expected to have anti-tumoral activity in pediatric preclinical cancer models and were associated with a more favorable clinical outcome in the present study. This warrants further studies including prospective evaluation of baseline cytokine profile as a predictive biomarker for HDACi tolerability and/or response in certain subpopulations like HGG. The first pediatric studies are in preparation and will validate the potential predictive role of the baseline cytokine profile (NCT03838042).

## Methods

### Eligibility criteria

Children and adolescents (3–18 years) with relapsed or therapy-refractory solid tumors (including brain tumors), lymphoma, or leukemia following standard first-line or relapse protocols in pediatric oncology were eligible. Reference-confirmed diagnosis by one of the pathological, radiological, or study reference centers recognized by the Society for Pediatric Oncology und Hematology (GPOH) in Germany was required. No other simultaneous anti-neoplastic treatment or radiotherapy during the study and 2 weeks before enrolment was allowed. Other eligibility criteria included adequate general condition (Lansky Score > 50%) and life expectancy > 3 months, liver enzymes (ALT or AST) < ×5 upper limit of normal reference value, bilirubin and creatinine < ×3 upper limit of normal reference value, solid tumors: leukocytes > 2000/μl, thrombocytes > 50.000/μl and adequate bone marrow function to permit evaluations of hematopoietic toxicity, no CTCAE grade 3 or 4 toxicity from previous treatments (no persistent CTCAE ≥ grade 3 toxicity from previous treatments), normal ECG. For solid tumors (including brain tumors), measurable disease activity according to Response Evaluation Criteria in Solid Tumors (RECIST) version 1.1 was required. Written informed consent of the legal representatives and the patient (if the patient was able to understand the study situation and to give consent) was mandatory. Women with childbearing potential had to agree to use adequate contraception or to abstain from heterosexual activity throughout the study, starting with Visit 1, and sexually active male patients had to agree to use an adequate method of contraception for the duration of the study. Patients were excluded if they had a history of deep vein thrombosis or pulmonary embolism, were pregnant or lactating, and used concomitant treatments and/or anti-neoplastic treatment such as chemotherapy, immune therapy, and differentiation therapy, other targeted therapy, or received radiotherapy. The use of valproic acid as prior antiepileptic therapy was allowed with a 14-day washout period. Other exclusion criteria included prior exposure to HDACi, known active HBV, HCV, or HIV infection, the use of concomitant treatments such as amber (*Hypericum perforatum*), plant extracts, vitamins, and other anti-oxidative compounds, participation in other clinical trials or observation period of competing trials, respectively, unable to swallow vorinostat suspension or capsules, and use of coumarin-derivative anticoagulants and any other medication which could accentuate known dose-dependent adverse effects of the study drug, for instance bone marrow depression or QT-prolongation.

### Study design and treatment

The study design was a single-arm, multi-center phase I/II clinical trial with two phases: Phase I was an intra-patient dose (de)escalation period until an individual maximum tolerable dose (MTD) was reached. The vorinostat dose was escalated with increments of 50 mg/m^2^/day every week until dose limiting toxicity (DLT) occurred or a maximum dose of 580 mg/m^2^/day was reached (Additional file 4). The maximum dose was chosen based on early phase I adult dose escalation experience demonstrating that this was the highest tolerable dose, maximum drug exposure/on target activity, and clinical responses seen in individual cases [20]. A DLT was defined as grade 3 or 4 toxicity according to a selection of Common Toxicity Criteria for Adverse Events (CTCAE) (V4.0) and judged by the investigator as definitely, probably, or possibly related to the study drug. The starting dose was 180 mg/m^2^/day and the minimum dose was 30 mg/m^2^/day. A dosing nomogram was used to minimize interpatient dosing variability.

In case of a DLT, vorinostat was discontinued until toxicity had declined to grade 2 or less and treatment could then be resumed at the previous dose without grade 3 or 4 toxicity (reduction by 50 mg/m^2^/day). This dose was defined as the individual MTD. If a DLT already occurred at the starting dose, vorinostat was discontinued until toxicity had declined to at least grade 2 or less and treatment was continued with 130 mg/m^2^/day (Additional file 1). De-escalation was done in steps of 50 mg/m^2^/day until a minimal dose of 30 mg/m^2^/day was reached. If de-escalation resulted in a dose < 30 mg/m^2^/day, treatment was discontinued. Upon reaching her or his individual MTD, a patient seamlessly entered phase II of the protocol.

In phase II, during which vorinostat was administered at the individual MTD, the same de-escalation rules were applied. During phase II, disease assessments were performed every 3 months, starting 3 months after reaching individual MTD. If treatment was discontinued for any reason during phase II, treatment was prolonged thereafter to provide a minimal treatment window of 3 months before measuring response. Patients without progressive disease continued the therapy at their individual MTD until disease progression (but no longer than 9 months). In case of clinical benefit, treatment could be continued on an individual basis at the discretion of the investigator. Toxicities were graded according CTCAE V4.0.

Vorinostat was provided as 100-mg capsules (hard gelatin capsules) or suspension 50 mg/ml (capsules dispersed in OraPlus/OraSweet), both for once daily oral administrations with food. Capsules were supplied by Merck, Sharp & Dome (MSD) Germany, and drug distribution and suspension preparation were performed by the Heidelberg University Hospital Pharmacy Department.

### Study end points and assessments

The primary end point was to determine a safe dose recommendation (SDR), defined as the highest dose with a DLT in no more than 1/50 patients. Secondary end points included PK analyses at the first dose level and at the individual MTD, best overall response rate (combining CR, PR, and SD) after 3 months treatment at individual MTD and every 3 months thereafter, determination of duration of response and assessment of feasibility and safety of the intra-patient dose escalation design and treatment at MTD in the phase II part of the trial thereafter. Cytokine profiles as potential biomarker for response prediction to vorinostat were determined in an exploratory manner.

Pretreatment evaluations included medical history, physical examination, ECG, ß-hCG in urine pregnancy test (if applicable), and complete blood count including differential, serum electrolytes liver, and renal function tests. Tumor markers were determined if appropriate. During phase I and the first 3 months of phase II (until the first response evaluation), history, physical examination, and laboratory studies were obtained weekly. If patients continued treatment beyond the first response evaluation in phase II (i.e., not showing PD), evaluations were performed every 2 weeks. After end of treatment, patients were followed-up for 3 months according the same scheme. In addition, an ECG was performed during phase I on day 8, 15, and at the time of reaching the individual MTD. Disease evaluations were performed at baseline, after 3 months of treatment at individual MTD, and every 3 months thereafter, using MRI (solid tumors, brain tumors, lymphomas), MIBG scan (neuroblastoma), or bone marrow evaluation (leukemia). Responses were reported using RECIST version 1.1 (solid tumors, brain tumors, lymphomas) [42–44] or the International Neuroblastoma Response Criteria (INRC) (neuroblastoma) [45], by central review. Bone marrow aspirates were evaluated in a central lab together with peripheral blood differential blood counts.

### Pharmacokinetic studies

PK evaluation was performed at day 8 after start treatment, at the time of reaching the individual MTD, and 3 months thereafter (at the time of the first response evaluation). Samples (2-mL citrate-blood) were collected before, 0.5, 1, 1.5, 2, 4, 6, and 8 h after oral vorinostat administration. Vorinostat plasma concentrations were quantified according to previously published methods [46].

The assays were validated according to the common FDA and EMA guidelines on bioanalytical method validation. Lower limit of quantification of vorinostat was 11.0 ng/mL. The calibrated vorinostat range was 11.0–1100 ng/mL with correlation coefficients > 0.99. The overall accuracy varied between − 6.7% and + 3.8% and the overall precision ranged from 3.2 to 6.1%.

### Biological analyses

Biomarker evaluation (cytokine profiles) was performed at baseline, day 8, at the time of reaching the individual MTD, and 3 months thereafter (3 mL citrate blood). Cytokine measurements in plasma samples were performed using multiplex assay (“Luminex”) as described [47]. *n* = 27 different cytokines/chemokines were analyzed in one sample using Bio-Plex ProTM Human Cytokine 27-plex Assay (cat. no. 244 M500KCAF0Y, Bio-Rad) according to the manufacturer’s protocol. Molecular profiling was done by DNA methylation array [48] and custom gene panel sequencing [49] in patients with long-term disease stabilization if archival tumor material was available.

### Statistical analyses

#### Safety and efficacy

The justification for the sample size was based on accuracy requirements for the SDR. Fifty pediatric patients were to be included in the trial. If DLT was observed at a given dose *d* in no more than 1/50 patients (this defines the SDR for routine application), then the upper bound of the 95% confidence interval for the true rate *r* of DLT at this dose was ≤ 10.65%. The DLT associated with the starting dose was to be continuously monitored using a Bayesian criterion with a non-informative prior and a binomial-beta model for the DLT rate *r*. If, for the second and following patients, the posterior probability that *r* > 10% was 95% or higher, the starting dose used for the following patients had to be lowered by 50 mg/m^2^. This decision process was repeated, i.e., it was applied to the lowered starting dose in an analogous way. If, during dose escalation, a patient experienced drug-related life-threatening symptoms or death, dose escalation for the following patients had to be stopped one dose step below this toxic dose.

Apart from the estimation of the SDR and the treatment response rates with exact two-sided 95% CI according to Clopper-Pearson [50], the statistical analysis was explorative and mainly descriptive. Standard methods for survival analysis (e.g., Kaplan-Meier estimates of the survival curves, Greenwood’s formula for estimating the standard error of event rates) were used for the analysis of time-to-event endpoints. The main analysis based on the safety analysis set included all enrolled patients who had taken at least one dose of trial treatment. Efficacy analysis included all patients who had completed the escalation/de-escalation scheme and started with the individual therapy at the MTD.

All adverse events were summarized, using MedDRA preferred terms. Serious adverse event presentations were derived from a separate, centralized, adverse event monitoring database that was continuously updated based on rapidly communicated reports from the investigators to the sponsor.

### Pharmacokinetic analyses

PK parameters were calculated by non-compartmental analysis using plasma concentrations of vorinostat (C_max_/D, T_Max_, AUC/D, T_1/2_, clearance Cl/F, and distribution Volume V_z_/F). The AUCs were calculated by mixed log-linear model. Following exploratory aims to predict progression free survival (PFS) and overall survival (OS), an optimal cutpoint in the continuous PK parameter distributions of C_max_ and AUC/D was determined by the minimum *p* value approach based on the log-rank test [51]. Graphical and statistical analysis was performed with Graphpad Prism 7.0 (Graphpad Software Inc., La Jolla, CA, USA), Kinetica 5.0 (Thermo Fisher Scientific, Waltham, MA, USA), and the R software/environment version 3.5.1 (R Foundation for Statistical Computing, Vienna, Austria).

### Biomarker evaluation (cytokine profiles)

Values below the lowest standard curve value were extrapolated. Values below the assay detection limit (out of range, OOR), i.e., below extrapolated values, were treated as left-censored. IL5 and IL15 were almost only OOR and therefore not informative, and excluded from further analysis. Hierarchical clustering of samples based on baseline concentrations was performed after Gehan’s U-score transformation of ranked cytokine levels was applied. L1-penalized logistic regression was used to discard strongly correlated cytokines for cluster assignment. Log-rank test was used to compare prognosis between clusters. Spearman’s correlation coefficient was used to assess pairwise correlation between cytokines. Mean values were estimated using Regression on Order Statistics with the R package NADA [52].

## Supplementary information

**Additional file 1: Figure S1. a** Linear correlation C_max_ (ng/mL) – Dose (mg/m^2^/d). **b** Concentration of vorinostat in plasma according to dose level.

**Additional file 2: Figure  S2. a** Plot of pairwise correlations (Spearman) of baseline concentrations of individual cytokines. **b** Heatmap of baseline concentrations (Gehan u-score) of a selection of 9 cytokines. **c** Longitudinal analysis of mean values of cytokines, separated by MTD groups. **d** Longitudinal analysis of mean values of cytokines, separated by best response groups.

**Additional file 3: Figure S3. **Cluster discrimination on single factor levels. **a** Receiver operating characteristic curve (ROC) for IL8 discriminating “high” and “low” (cluster 1), showing high correlation of sensitivity and specificity resulting in good predictivity. **b** Boxplot of IL8 cytokine concentrations in groups “high” and “low” (cluster 1). **c** and **d**: boxplots of cytokines IL9 (c) and MIP1b (d) showing additional discrimination of clusters “high”, “intermediate” and “low” (cluster 2).

**Additional file 4:** **Table S1.** Dose levels and DLTs.

**Additional file 5:** **Table S2.** Summary of safety data.

**Additional file 6:** PK parameters (± SD). Medians and means of all dose levels.

## Data Availability

Datasets supporting the conclusions of this article are included within the article and its additional files. In addition, the datasets used and/or analyzed during the current study are available from the corresponding author on reasonable request.
