# Supplementary material for: Phase I/II intra-patient dose escalation study of vorinostat in children with relapsed solid tumor, lymphoma, or leukemia
Source: Clin Epigenetics. 2019 Dec 10;11:188. doi: 10.1186/s13148-019-0775-1 (PMC6902473; doi:10.1186/s13148-019-0775-1)
Supplement: Supplementary file 1 — Additional file 1: Figure S1. a Linear correlation Cmax (ng/mL) – Dose (mg/m2/d). b Concentration of vorinostat in plasma according to dose level. [file 13148_2019_775_MOESM1_ESM.pptx]

## Slide 1
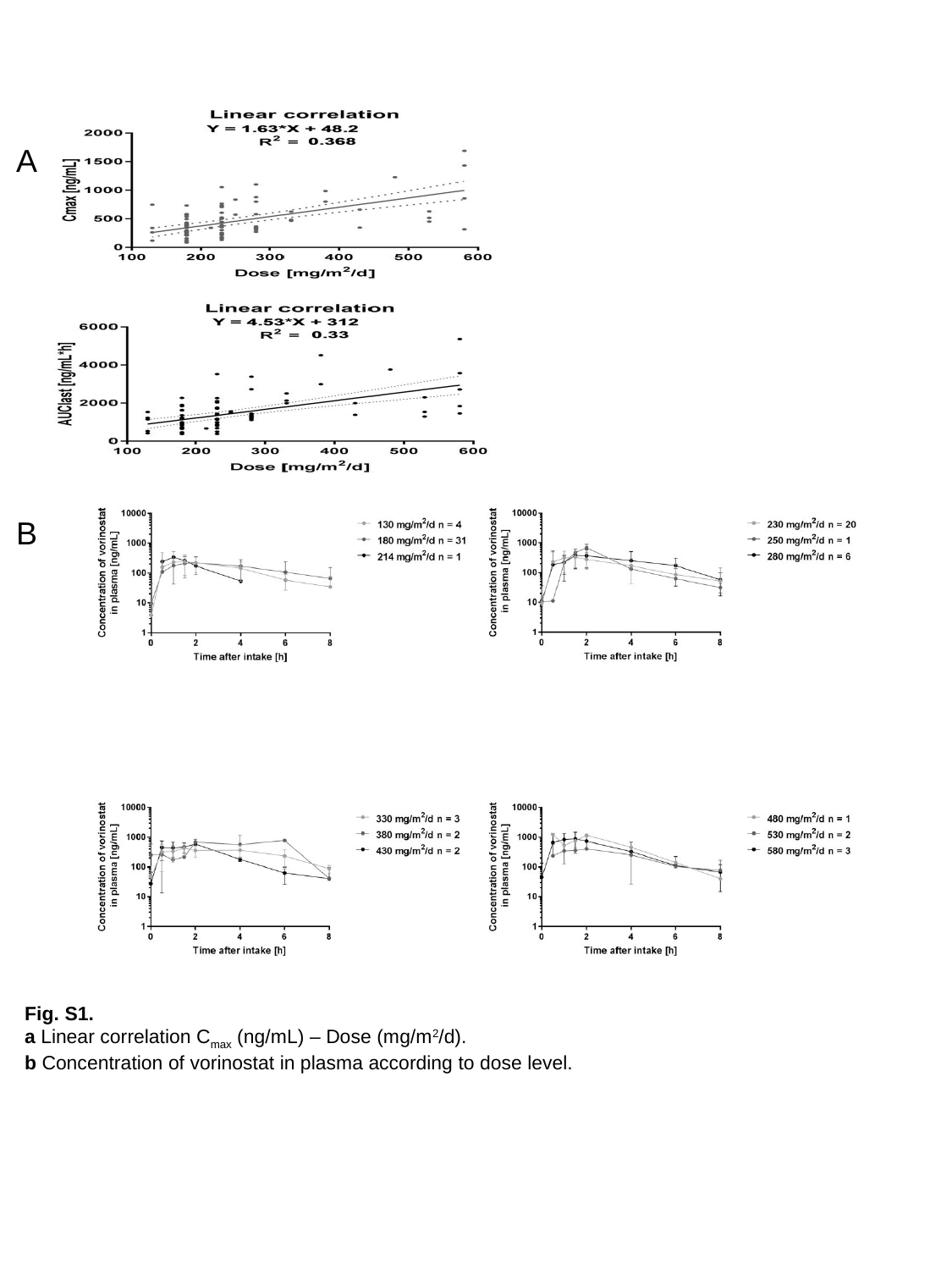

A
B
Fig. S1.
a Linear correlation Cmax (ng/mL) – Dose (mg/m2/d).
b Concentration of vorinostat in plasma according to dose level.
