# Supplementary material for: Phase I/II intra-patient dose escalation study of vorinostat in children with relapsed solid tumor, lymphoma, or leukemia
Source: Clin Epigenetics. 2019 Dec 10;11:188. doi: 10.1186/s13148-019-0775-1 (PMC6902473; doi:10.1186/s13148-019-0775-1)
Supplement: Supplementary file 2 — Additional file 2: Figure S2. a Plot of pairwise correlations (Spearman) of baseline concentrations of individual cytokines. b Heatmap of baseline concentrations (Gehan u-score) of a selection of 9 cytokines. c Longitudinal analysis of mean values of cytokines, separated by MTD groups. d Longitudinal analysis of mean values of cytokines, separated by best response groups. [file 13148_2019_775_MOESM2_ESM.pptx]

## Slide 1
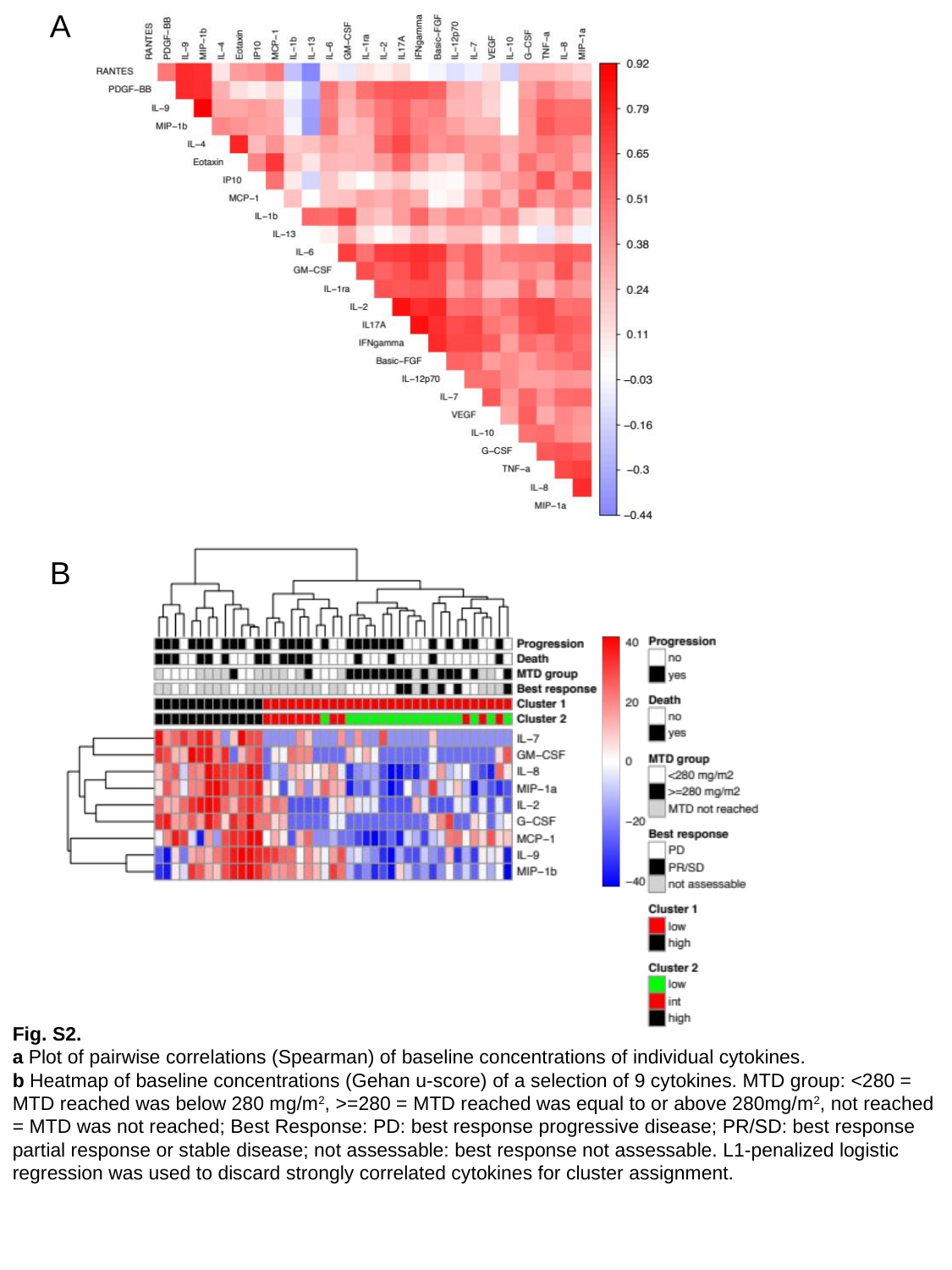

A
B
Fig. S2.
a Plot of pairwise correlations (Spearman) of baseline concentrations of individual cytokines.
b Heatmap of baseline concentrations (Gehan u-score) of a selection of 9 cytokines. MTD group: <280 = MTD reached was below 280 mg/m2, >=280 = MTD reached was equal to or above 280mg/m2, not reached = MTD was not reached; Best Response: PD: best response progressive disease; PR/SD: best response partial response or stable disease; not assessable: best response not assessable. L1-penalized logistic regression was used to discard strongly correlated cytokines for cluster assignment.

## Slide 2
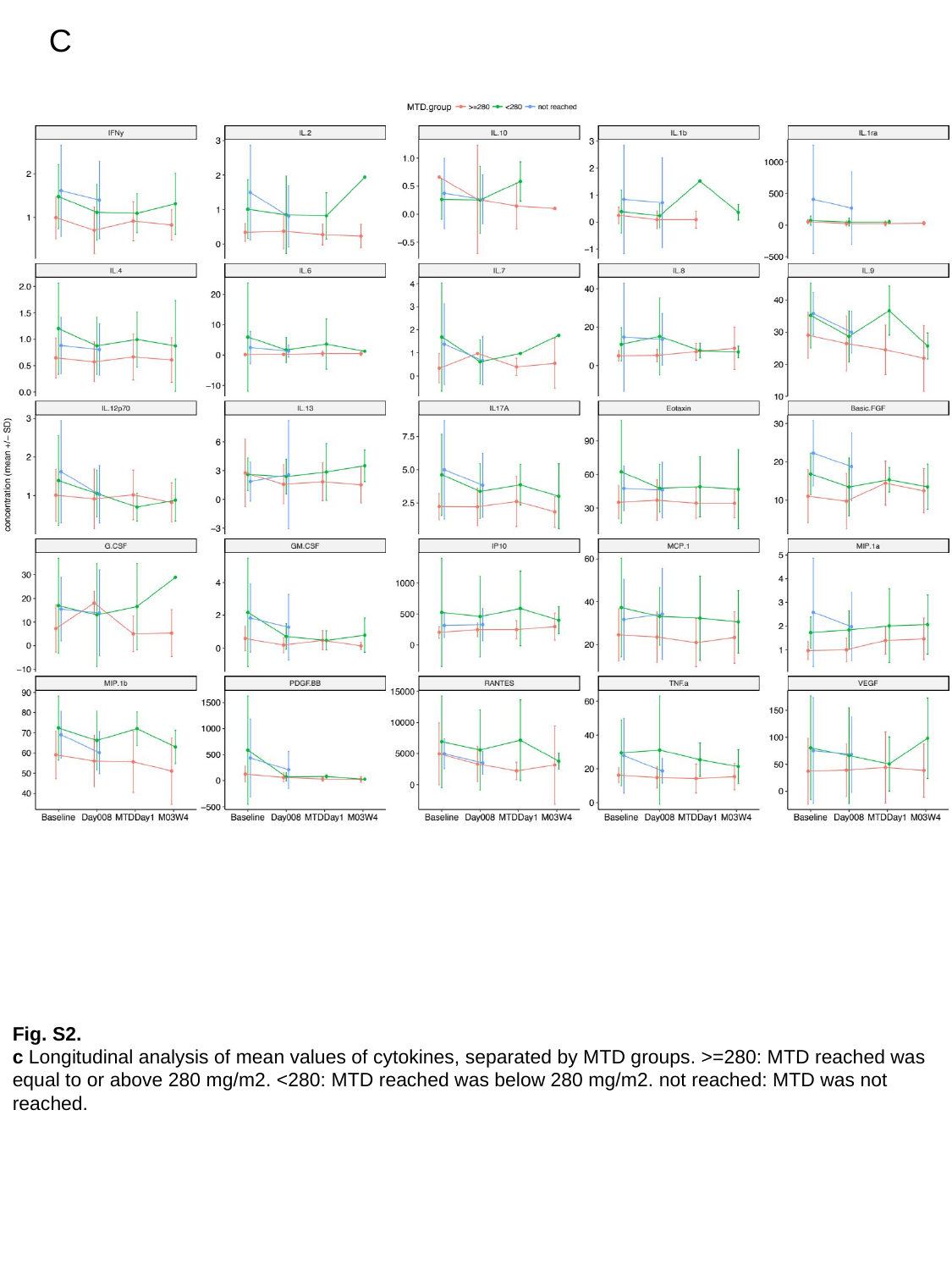

C
Fig. S2.
c Longitudinal analysis of mean values of cytokines, separated by MTD groups. >=280: MTD reached was equal to or above 280 mg/m2. <280: MTD reached was below 280 mg/m2. not reached: MTD was not reached.

## Slide 3
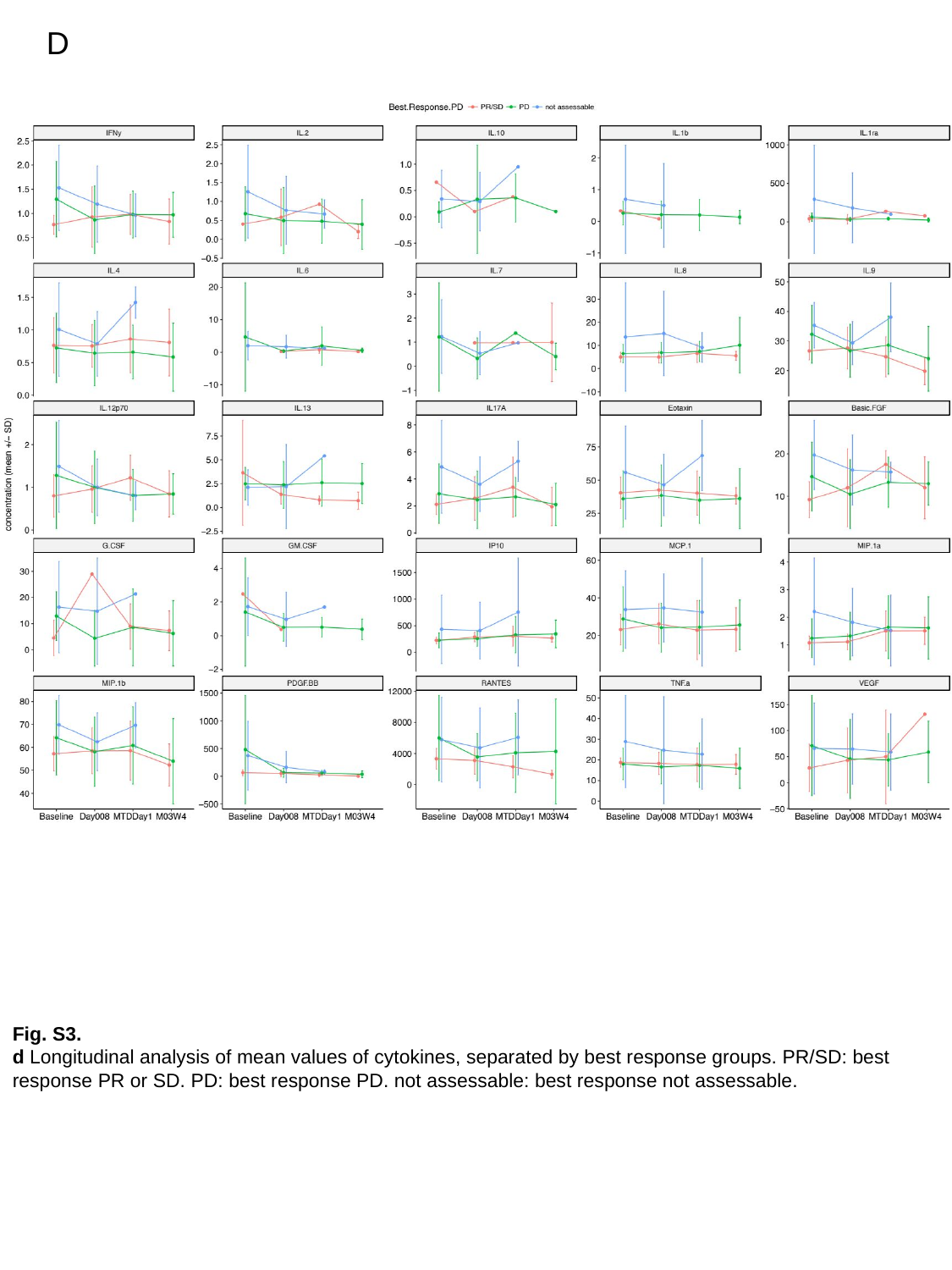

D
Fig. S3.
d Longitudinal analysis of mean values of cytokines, separated by best response groups. PR/SD: best response PR or SD. PD: best response PD. not assessable: best response not assessable.
