# Supplementary material for: Phase I/II intra-patient dose escalation study of vorinostat in children with relapsed solid tumor, lymphoma, or leukemia
Source: Clin Epigenetics. 2019 Dec 10;11:188. doi: 10.1186/s13148-019-0775-1 (PMC6902473; doi:10.1186/s13148-019-0775-1)
Supplement: Supplementary file 3 — Additional file 3: Figure S3. Cluster discrimination on single factor levels. a Receiver operating characteristic curve (ROC) for IL8 discriminating “high” and “low” (cluster 1), showing high correlation of sensitivity and specificity resulting in good predictivity. b Boxplot of IL8 cytokine concentrations in groups “high” and “low” (cluster 1). c and d: boxplots of cytokines IL9 (c) and MIP1b (d) showing additional discrimination of clusters “high”, “intermediate” and “low” (cluster 2). [file 13148_2019_775_MOESM3_ESM.pptx]

## Slide 1
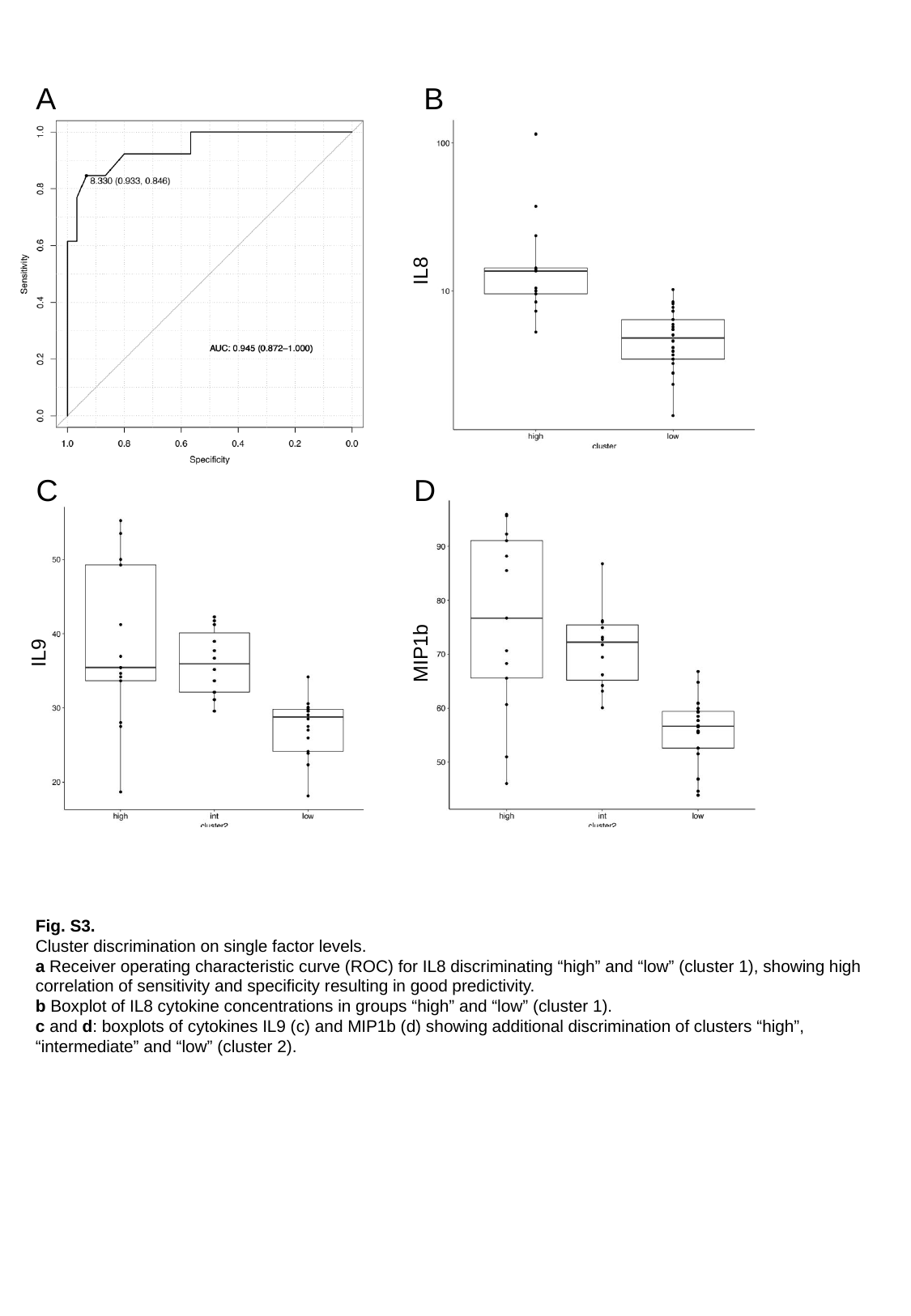

A
B
IL8
C
D
IL9
MIP1b
Fig. S3.
Cluster discrimination on single factor levels.
a Receiver operating characteristic curve (ROC) for IL8 discriminating “high” and “low” (cluster 1), showing high correlation of sensitivity and specificity resulting in good predictivity.
b Boxplot of IL8 cytokine concentrations in groups “high” and “low” (cluster 1).
c and d: boxplots of cytokines IL9 (c) and MIP1b (d) showing additional discrimination of clusters “high”, “intermediate” and “low” (cluster 2).
