# Supplementary material for: Phase I/II intra-patient dose escalation study of vorinostat in children with relapsed solid tumor, lymphoma, or leukemia
Source: Clin Epigenetics. 2019 Dec 10;11:188. doi: 10.1186/s13148-019-0775-1 (PMC6902473; doi:10.1186/s13148-019-0775-1)
Supplement: Supplementary file 4 — Additional file 4: Table S1. Dose levels and DLTs. [file 13148_2019_775_MOESM4_ESM.docx]

Table S1. Dose levels and DLTs.

| **Dose level** | **Dosis**  **(mg/m^2^/d)** | **Patients with DLT**  **No. (%; 95% confidence interval)** |
| --- | --- | --- |
| -3 | 30 | - |
| -2 | 80 | - |
| -1 | 130 (SDR) | 1 (2; 0.1 – 10.6) |
| Start | 180 | 10 (20; 10.0 – 33.7) |
| 1 | 230 | 14 (28; 16.2 – 42.5) |
| 2 | 280 | 15 (30; 17.9 – 44.6) |
| 3 | 330 | 10 (20 ; 10.03 – 33.72) |
| 4 | 380 | 5 (10; 3.3 – 21.8) |
| 5 | 430 | 6 (12; 4.5 – 24.3) |
| 6 | 480 | 4 (8; 2.2 – 19.2) |
| 7 | 530 | 4 (8; 2.2 – 19.2) |
| 8 | 580 | 5 (10; 3.3 – 21.8) |

Planned dose levels and patients with DLTs at the respective dose levels (safety population, n=50). Irrespective of the number of DLTs, all patients were only counted once per dose level. Since patients could show a DLT again after dose de-escalation for a DLT, individual patients could experience several DLTs at different dose levels. Confidence interval according to Clopper-Pearson.
