# Supplementary material for: Phase I/II intra-patient dose escalation study of vorinostat in children with relapsed solid tumor, lymphoma, or leukemia
Source: Clin Epigenetics. 2019 Dec 10;11:188. doi: 10.1186/s13148-019-0775-1 (PMC6902473; doi:10.1186/s13148-019-0775-1)
Supplement: Supplementary file 5 — Additional file 5: Table S2. Summary of safety data. [file 13148_2019_775_MOESM5_ESM.docx]

Table S2. Summary of safety data (safety population, n=50).

|  | **Patients with all causalities AE**  **No. (%)** | **Patients with treatment related AE**  **No. (%)** |
| --- | --- | --- |
| AE | 49 (98) | 46 (92) |
| SAEs | 19 (38) | 6 (12) |
| Severe AEs (CTCAE grade 3/4) | 48 (96) | 42 (84) |
| AE leading to treatment discontinuation | 9 (18) | 6 (12) |
| AE leading to dose reduction or temporary treatment discontinuation | 38 (76) | 35 (70) |
